# Highly Efficient Oxygen Electrode Obtained by Sequential Deposition of Transition Metal-Platinum Alloys on Graphene Nanoplatelets

**DOI:** 10.3390/ma16093388

**Published:** 2023-04-26

**Authors:** Dušan Mladenović, Elif Daş, Diogo M. F. Santos, Ayşe Bayrakçeken Yurtcan, Biljana Šljukić

**Affiliations:** 1 University of Belgrade, Faculty of Physical Chemistry, Studentski Trg 12-16, 11158 Belgrade, Serbia; dusan.mladenovic@ffh.bg.ac.rs; 2 Department of Physics, Atatürk University, 25240 Erzurum, Turkey; elif.das@atauni.edu.tr; 3 Center of Physics and Engineering of Advanced Materials, Laboratory for Physics of Materials and Emerging Technologies, Chemical Engineering Department, Instituto Superior Técnico, Universidade de Lisboa, 1049-001 Lisbon, Portugal; diogosantos@tecnico.ulisboa.pt; 4 Department of Chemical Engineering, Atatürk University, 25240 Erzurum, Turkey; abayrakceken@atauni.edu.tr

**Keywords:** alloy nanoparticles, graphene nanoplatelets, oxygen reduction reaction, oxygen evolution reaction, bifunctional electrocatalyst

## Abstract

A set of platinum (Pt) and earth-abundant transition metals (M = Ni, Fe, Cu) on graphene nanoplatelets (sqPtM/GNPs) was synthesised via sequential deposition to establish parallels between the synthesis method and the materials’ electrochemical properties. sqPtM/GNPs were assessed as bifunctional electrocatalysts for oxygen evolution (OER) and reduction (ORR) reactions for application in unitised regenerative fuel cells and metal-air batteries. sqPtFe/GNPs showed the highest catalytic performance with a low potential difference of ORR half-wave potential and overpotential at 10 mA cm^−2^ during OER, a crucial parameter for bifunctional electrocatalysts benchmarking. A novel two-stage synthesis strategy led to higher electrocatalytic performance by facilitating the reactants’ access to the active sites and reducing the charge-transfer resistance.

## 1. Introduction

In light of new technologies and growing energy demands, low-cost and environmentally acceptable energy conversion devices are essential. At present, Li-ion batteries (LIBs) have increased in popularity and become one of the primary devices for storing electrical energy for everyday use. Although LIBs have many advantages [1], there are also considerable disadvantages concerning the toxicity of the organic electrolyte and its flammability, making them relatively unsafe to use. An alternative is a fuel cell device that converts the chemical energy of reacting species into electricity. There are many types of fuel cells, and their effectiveness largely depends on the type of electrolyte they use. Furthermore, there are fuel cells that can operate in two separate modes, one in which they produce energy by burning fuel (usually hydrogen) and oxygen, and the second in which they can produce hydrogen and oxygen from water (electrolysis mode). These fuel cells are called unitised regenerative fuel cells (URFCs).

The main limiting factor for greater exploitation and mass production of fuel cells is the sluggish kinetics of the oxygen reduction reaction (ORR). Namely, platinum (Pt) is the material of choice for ORR catalysis. ORR on Pt proceeds via the so-called direct 4e^−^ mechanism, where 4e^−^ are exchanged in the elementary step of reaction and oxygen is reduced directly to water. Most catalysts proceed via the 2e^−^ mechanism, with at least one intermediary species. For URFCs, the criteria for choosing the oxygen electrode catalyst are even greater because the oxygen evolution reaction (OER), another efficiency-limiting reaction, takes place in the electrolysis mode.

Catalysts’ synthesis methods have significant importance in their catalytic activities. The supercritical carbon dioxide deposition technique has several advantages over conventional catalyst preparation techniques. It involves the dissolution of an organometallic precursor in a supercritical carbon dioxide medium and adsorption of the support material. The metallic form of the precursor over the support material can be achieved by in situ or ex-situ thermal treatment methods [2]. Supported alloy catalysts can be synthesised by following the simultaneous [3] or sequential [4] supercritical carbon dioxide deposition techniques, resulting in uniform compositions and highly dispersed catalysts. In the simultaneous deposition, both metal precursors are adsorbed by the support material and then reduced to their metallic form. In the sequential deposition, one precursor is first adsorbed by the support material and reduced to its metallic form. Then, the second precursor adsorption and reduction result in the alloy catalyst. Changes in the positioning of the nanoparticles (NPs) over the support material in simultaneous or sequential deposition techniques may result in changes in the catalytic properties, such as morphology, size, stability, and electrochemical activity. In previous research, three PtNi, PtFe, and PtCu alloys on carbon material were synthesised via a simultaneous deposition technique [5]. Results showed that catalysts with simultaneously deposited Pt and Fe NPs reduced oxygen to water by a direct 4e^−^ pathway, with a higher diffusion-limited current density than the samples with PtNi and PtCu NPs deposited on graphene nanoplatelets (GNPs). Furthermore, PtFe catalysts showed promising results in OER catalysis, indicating potential application for URFCs cathodes. Carbon aerogel-supported and Vulcan-supported PtCu catalysts were synthesised by sequential and simultaneous supercritical carbon dioxide deposition techniques [6]. The researchers used copper (II) trifluoroacetylacetonate (Cu(tfa)_2_) as the copper precursor and obtained lower second metal loadings with sequential carbon dioxide deposition. PtCu/Vulcan catalysts synthesised by simultaneous and sequential deposition techniques showed two and five times higher specific activity, respectively, than commercial Pt/C, which was attributed to the Cu-rich shell/PtCu core NP morphology. The performance of proton-exchange membrane (PEM) fuel cells using the sequentially deposited catalysts was superior to the simultaneously deposited ones [4,5]. Moreover, higher second metal loading values were obtained for sequentially deposited bimetallic catalysts using copper (II) hexafluoroacetylacetonate hydrate (C_10_H_2_CuF_12_O_4_·xH_2_O) as the copper precursor [5].

The arrangement of the higher activity Pt catalyst, when compared to the lower activity second metal, over the support material can be provided by first including Pt and then the second metal, resulting in the sequential deposition. This may provide the reactants better access to the Pt active sites of the catalysts. The operating conditions, support material, and metal precursor types each strongly affect the properties of the synthesised catalysts. A set of three alloy (PtNi, PtFe, and PtCu) catalysts on GNPs were synthesized herein via the sequential deposition method to establish potential parallels between the synthesis and the electrochemical properties of the electrocatalysts.

## 2. Materials and Methods

### 2.1. Preparation of the GNPs-Supported PtM Catalysts

**Scheme 1:**
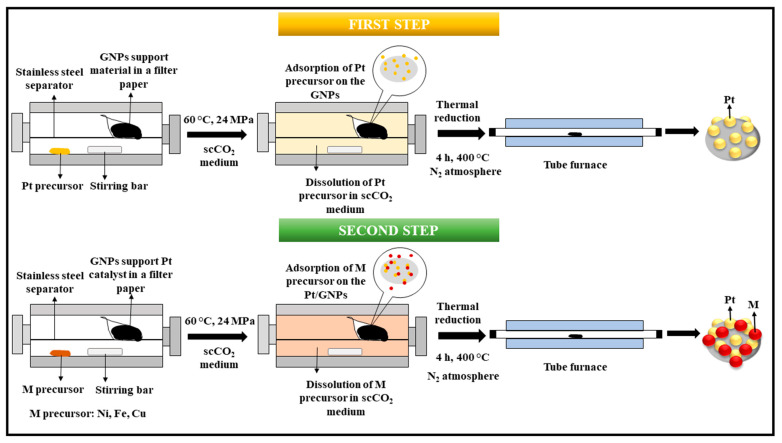
The synthesis procedure of GNPs-supported PtM catalysts.

Alloy nanoparticles (NPs) were grafted onto graphene nanoplatelets (GNPs, xGnP^®^ Grade C, XG Sciences, Lansing, MI, USA) via the sequential supercritical carbon dioxide (scCO_2_) deposition technique. The synthesis process was carried out in the scCO_2_ medium, involving two sequential steps, as shown in Scheme 1. In the first step, the primary metal (Pt) was deposited onto the GNPs by scCO_2_ under optimal operational conditions, according to earlier results [7]. Subsequently, the secondary metal (Ni, Fe, or Cu) was deposited onto the Pt/GNPs material. The deposition procedure is detailed in a recent study [4]. Namely, GNPs (0.1 g) and 1,5-dimethyl platinum cyclooctadiene (0.2 g) as the Pt organometallic precursor were placed in a filter-paper bag and heated to 60 °C in a high-pressure vessel. After reaching the set temperature, carbon dioxide (CO_2_) was fed to the vessel via a syringe pump. The vessel was pressurised up to 24 MPa to provide scCO_2_ conditions. These conditions were maintained for 24 h for the system to reach equilibrium. After 24 h, the obtained material was placed in a tube furnace, and the metal precursor was converted to the corresponding metal by heating at 400 °C for 4 h under N_2_ flow. This resulted in Pt NPs grafted onto GNPs (Pt/GNPs). The same sequence of steps was repeated to synthesise the sqPtM/GNPs catalysts, but Pt/GNPs (0.1 g) and the secondary metal precursor (0.2 g, nickel (II) hexafluoroacetylacetonate hydrate, copper (II) hexafluoroacetylacetonate hydrate or Fe (III) tris (2,2,6,6-tetramethyl-3,5-heptanedionate) were used instead of GNPs and the Pt precursor, respectively. The amounts of the corresponding precursors were determined by using the adsorption isotherms of the precursor onto the GNPs support [4].

### 2.2. Physical Characterization of the Catalysts

Inductively coupled plasma mass spectrometry (ICP-MS) measurements were carried out with an Agilent 7800 ICP mass spectrometer to determine the metal loading in each sqPtM/GNPs catalyst. Transmission electron microscopy (TEM) images were taken via Hitachi HighTech HT7700 to observe the distribution of metallic particles on the GNPs. X-ray photoelectron spectroscopy (XPS) measurements were taken using a Thermo Scientific K_α_ X-ray spectrometer to identify the materials’ respective compositions and surface oxidation states.

### 2.3. Electrode Preparation and Electrochemical Measurements

Catalytic inks were prepared with Nafion as a binder [8]. 10 µL of each catalyst ink was dripped onto a glass carbon rotating disc electrode (RDE, 0.19625 cm^2^). Working electrodes were then dried by blowing high-purity N_2_ over them.

Electrochemical experiments were performed in a standard three-electrode electrochemical cell with Pt mesh and saturated calomel electrodes (SCEs) as counter and reference electrodes, respectively, using a Gamry Interface 1010 galvanostat/potentiostat. For RDE experiments, the rotation rate of the working electrode was controlled via the Gamry rotator (Gamry RDE710 Rotating Electrode). The atmosphere in the cell was controlled by bubbling high-purity gases (O_2_ or N_2_, Messer, 99.9995 vol.%) before every measurement.

The stability of all catalysts for ORR was studied for 4 h in chronoamperometry mode with constant O_2_ bubbling. In contrast, stability for OER was investigated using the ARBIN instrument for 10 h in chronoamperometry mode at a potential of 1.7 V vs. RHE. OER tests were performed in the so-called “switching mode”, where a 120 s pulse of ORR potential was applied, followed by a 30 s OER pulse. By applying ORR potential first, the electrode surface was cleaned of gas bubbles formed in OER mode, and in addition, the real-life operating environment of URFCs was simulated.

## 3. Results & Discussion

### 3.1. Catalysts Characterization

**Figure 1:**
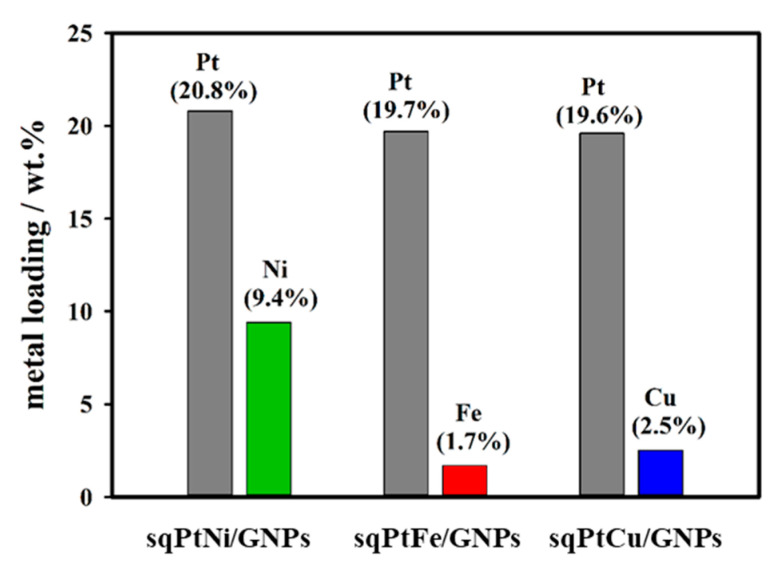
ICP-MS results of sqPtM/GNPs catalysts.

PtM/GNPs catalysts were analysed using the ICP-MS technique to determine the relative metal compositions, shown in Figure 1. The results reveal that all catalysts contain similar amounts of platinum (ca. 20 wt.% of Pt). The amount of secondary metal deposited on the surface of the Pt/GNPs catalyst is considerably lower, suggesting a limitation for the deposition of Ni, Fe, or Cu on the surface of platinum.

**Figure 2:**
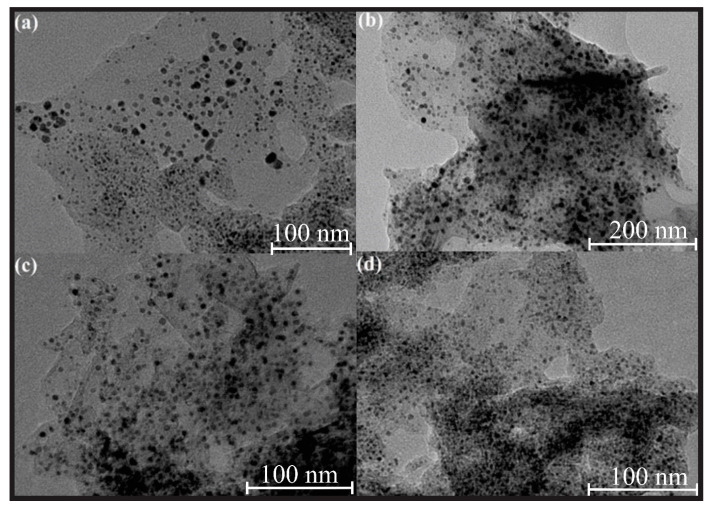
TEM images of (**a**) sqPtNi/GNPs, (**b**) sqPtFe/GNPs, (**c**) sqPtCu/GNPs, and (**d**) Pt/GNPs catalysts.

XRD analysis of the prepared materials showed that the bimetallic NPs have disordered alloy crystal structures [4]. Figure 2 shows representative TEM images of sqPtM/GNPs catalysts, where highly dispersed sphere-like particles (3–5.5 nm size range) are visible in each sample. After decorating the secondary metal on monometallic Pt/GNPs, the average particle size for each bimetallic sqPtM/GNPs catalyst increased slightly. This demonstrates the deposition of the secondary metals (Ni, Fe, and Cu) on Pt/GNPs. The EDX analysis of the materials confirmed that they comprise the corresponding metals [4]. These findings were further verified by elemental mapping that showed the presence of C, Pt and secondary elements (Ni, Fe, and Cu) in the structure [4].

**Figure 3:**
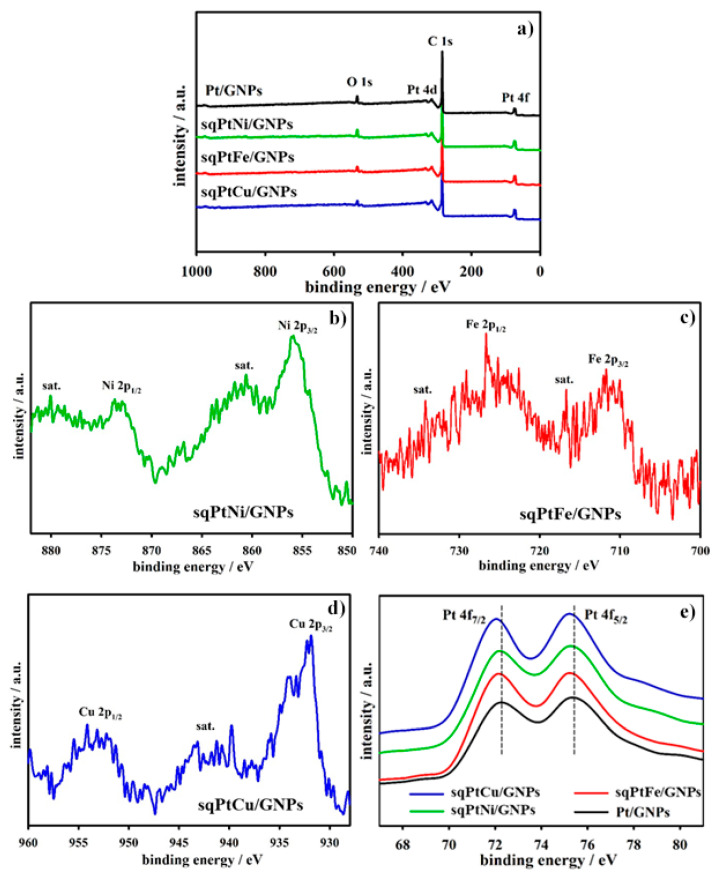
(**a**) Survey scan spectra of Pt/GNPs and sqPtM/GNPs catalysts. High-resolution wide-scale spectra of the (**b**) Ni, (**c**) Fe, and (**d**) Cu elements. (**e**) Pt 4f high-resolution XPS spectra of the Pt/GNPs [1] and sqPtM/GNPs catalysts.

XPS was used to analyse the prepared catalysts’ surface composition and oxidation states. Figure 3 shows the representative XPS survey spectra of the Pt/GNPs, sqPtNi/GNPs, sqPtFe/GNPs, and sqPtCu/GNPs catalysts. Four predominant peaks are present in the survey scan spectrum for all catalysts, attributed to the C 1s, O 1s, Pt 4f, and Pt 4d levels. However, no distinct peak was observed for Ni, Fe, and Cu metals. This can be explained by a low amount of secondary metal in the structure. To clarify this point, high-resolution XPS measurements for those regions were performed (Figure 3). Figure 3b shows the high-resolution wide-scale XPS spectra of Ni 2p of sqPtNi/GNPs alloy catalyst. The main Ni 2p_3/2_ and 2p_1/2_ peaks are observed at 855.7 and 873.2 eV, respectively, characteristic of oxidised Ni^2+^, with a small fraction at 852.3 eV, characteristic of Ni^0^. The peaks around 861.5 and 879.9 eV are respective satellite peaks. The presence of both characteristic and satellite peaks demonstrates the existence of Ni^2+^ in the sqPtNi/GNPs catalyst. The result is in agreement with the results of Samuel et al. [9]. They also reported that Ni 2p XPS spectra for NiFe_2_O_4_@NiNC had four peaks at the same binding energies. Similarly, Figure 3c demonstrates the high-resolution Fe 2p spectra along with two satellite peaks. The figure shows that the spectra contain a pair of peaks at binding energies of 711 eV (2p_3/2_) and 725 eV (2p_1/2_). These are attributed to the presence of Fe_2_O_3_ species in the sqPtFe/GNPs catalysts. The observed satellite peaks at around 717.5 and 733 eV confirm the existence of the Fe^3+^ states [10]. In addition to the Ni and Fe 2p spectra, the Cu 2p spectra were also distinctly displayed to confirm the successful formation of the sqPtCu/GNPs catalyst (Figure 3d). The figure shows two dominant peaks at 932.2 and 953 eV corresponding to the Cu 2p_3/2_ and Cu 2p_1/2_ levels. These peaks indicate the presence of Cu^+^ in the sample. Moreover, Cu^2+^ is also verified by the appearance of a shoulder peak at 934.1 eV for Cu 2p_3/2_, a satellite peak at 943.4 eV, and 954.8 eV for Cu 2p_1/2._ The obtained results indicate that Cu exists as Cu (I) and Cu (II), two distinct oxidation states in the sqPtCu/GNPs catalyst [11].

To explore the possibility of electronic interactions between platinum and secondary metals, high-resolution XPS spectra for the Pt 4f region were also investigated (Figure 3e). The XPS result shows that the Pt 4f binding energy of Pt^0^ species is shifted to lower peak values for the PtFe, PtNi, and PtCu alloy samples compared to the positions displayed by the Pt^0^ species of Pt/GNPs. The minimum magnitude of the shift is ca. 0.1 eV. The decrease in the Pt binding energy for the sqPtNi/GNPs, sqPtFe/GNPs, and sqPtCu/GNPs alloy catalysts relative to the Pt/GNPs suggests an electron transfer from the secondary metals to Pt, related to perturbed electronic interaction between Pt and Ni, Fe, or Cu atomic orbitals and, in turn, to their alloy formation. Recent studies have reported similar results for PtM (M = Fe, Co, Ni, Cu, and other transition metals) alloys [12,13,14,15]. Neergat and Rahul [12] studied unsupported core-shell CuPt NPs and reported a shift in binding energy to values lower than a Pt standard. They attributed this shift to lower binding energy to an electronic modification of surface Pt by the inner Cu core. He et al. [13] reported the synthesis of PtNi bimetallic nanoclusters (NCs) by a modified reduction method. They observed a measurable shift in the binding energy of PtNi NCs towards lower values than Pt NCs.

Prepared alloy catalysts exhibited thermal stability with decomposition temperatures (5 wt.% loss) exceeding a 200 °C atmosphere, which makes them highly useful for low-temperature fuel cells [4].

### 3.2. Electrochemical Characterization

**Figure 4:**
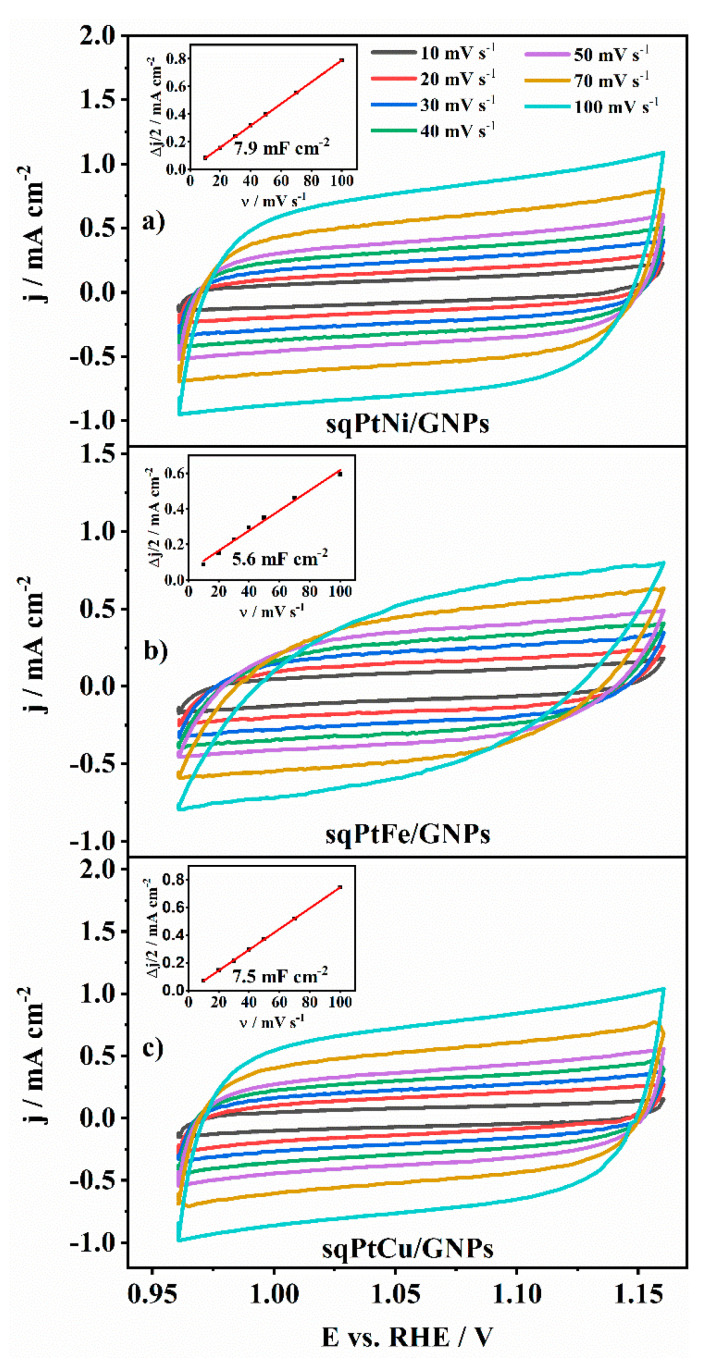
Cyclic voltammetry experiments in the double-layer region at different scan rates for (**a**) sqPtNi/GNPs, (**b**) sqPtFe/GNPs, and (**c**) sqPtCu/GNPs with the corresponding Δj = f(ν) plots in the inset.

The double-layer capacitance, C_dl_, directly proportional to the electrochemically active surface area (ECSA), represents the slope of Δj/2 (half the difference between non-faradaic anodic and cathodic current density, j_a_–j_c_) vs. scan rate, ν, plot. CVs for the three studied samples are shown in Figure 4, with the corresponding Δj/2 = f(ν) plot shown in the inset.

Similar C_dl_ values indicate a similar ECSA of the studied catalysts. Namely, C_dl_ of 7.9 mF cm^−2^, 5.6 mF cm^−2^, and 7.5 mF cm^−2^ were calculated for sqPtNi/GNPs, sqPtFe/GNPs, and sqPtCu/GNPs samples, respectively. Assuming that the specific capacitance of a flat surface is ≈40 µF cm^−2^ for 1 cm^2^ of real surface area, ECSA values of approximately 197 cm^2^, 140 cm^2^, and 187 cm^2^ were calculated for sqPtNi/GNPs, sqPtFe/GNPs, and sqPtCu/GNPs, respectively. The value of 40 µF cm^−2^ typically reported for a KOH electrolyte solution in McCrory et al. [16] was used for ease of comparison with literature data. However, it should be noted that specific capacitance depends on several factors (e.g., the oxidation state of the material) and can vary for each electrode [17,18].

An interesting trend was observed when comparing the data obtained herein to catalysts synthesised by simultaneous deposition [5]. Namely, when simultaneously deposited, the PtFe catalyst showed the highest C_dl_ value of 7.2 mF cm^−2^, followed by PtCu and PtNi (5.1 mF cm^−2^ and 4.5 mF cm^−2^, respectively) [5], the opposite of what is determined for sequentially deposited NPs. This indicates that the synthesis method substantially impacts catalyst morphology, and secondly, the synthesis method with better results varies for each PtM pair. For comparison, in the authors’ previous work, they also tested the Pt/GNPs catalyst, which showed a C_dl_ value of 8.2 mF cm^−2^, comparable to bimetallic catalysts [5], and commercial Pt/C catalysts (40 wt.% Pt), with lower C_dl_ of 3.1 mF cm^−2^ [8]. This indicates almost double (sqPtFe/GNPs) or even more than double (sqPtNi/GNPs and sqPtCu/GNPs) ECSA for the synthesised catalysts herein.

### 3.3. ORR Study

ORR has been extensively explored for many years as one of the main limiting factors in fuel cell operation. The sluggish kinetics of this reaction on most catalysts means that there is always a need for a new, improved and lower-cost material to replace Pt as the catalyst of choice for ORR in fuel cells.

**Figure 5:**
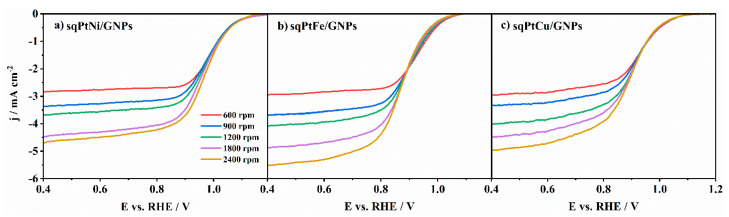
Linear scan voltammetry measurements of (**a**) sqPtNi/GNPs, (**b**) sqPtFe/GNPs, and (**c**) sqPtCu/GNPs at 20 mVs^−1^ and different rotation rates in 0.1 M KOH.

The activity of the three synthesised electrocatalysts towards ORR was tested by performing a series of linear scan voltammetry (LSV) measurements at different rotation rates in 0.1 M KOH at room temperature, shown in Figure 5. The acquired data allowed for calculating the Tafel slope (b), the number of exchanged electrons in the elementary step (n), half-wave potential (E_1/2_), diffusion-limited current density (j_d_), and kinetic current density (j_k_), as the key ORR parameters.

Diffusion-limited current density shows a well-defined plateau for all studied catalysts. Comparing LSVs at 1800 rpm, it was found that the sqPtFe/GNPs sample has the highest j_d_ value of −4.48 mA cm^−2^ compared to j_d_ values of −4.14 mA cm^−2^ and −3.99 mA cm^−2^ for the sqPtNi/GNPs and sqPtCo/GNPs catalysts, respectively. One could observe a similar trend when comparing the results obtained with PtM catalysts simultaneously deposited on GNPs [5]. Namely, PtFe simultaneously deposited on GNPs also showed the highest value of j_d_ (−4.65 mA cm^−2^). A lower j_d_ value of −3.71 mA cm^−2^ was determined for Pt/GNPs, while for the commercially acquired Pt/C catalyst (40 wt.% Pt) j_d_ was found to be −6.43 mA cm^−2^ [5]. These results are compared in Table 1. Although the commercial Pt/C catalyst reached the highest j_d_ value, the catalysts synthesized herein have approximately half of the Pt loading, making them substantially cheaper to produce.

**Figure 6:**
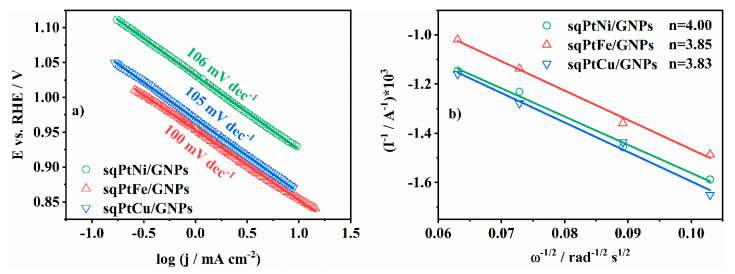
ORR (**a**) Tafel and (**b**) Koutecky-Levich plots of the studied catalysts.

From Tafel analysis of LSVs at 1800 rpm (Figure 6a), the lowest b value of 100 mV dec^−1^ was determined for ORR at sqPtFe/GNPs with slightly higher b values of 105 mV dec^−1^ and 106 mV dec^−1^ for sqPtCu/GNPs and sqPtNi/GNPs catalysts, respectively, as shown in Table 1.

Koutecky-Levich analyses (K-L) of LSVs recorded at different rotation rates enabled the determination of the number of electrons exchanged during ORR. The results of the K-L analysis are shown in Figure 6b. Plotted results represent straight lines indicating the first-order reaction. Accordingly, the number of exchanged electrons was calculated to be 3.85, 4.00, and 3.83 for sqPtFe/GNPs, sqPtNi/GNPs, and sqPtCu/GNPs electrocatalysts, respectively. Compared to the calculated values for the corresponding PtFe, PtNi, and PtCu NPs simultaneously deposited on GNPs (n values of 3.66, 3.93, and 3.61, respectively) [5], catalysts with NPs sequentially deposited on GNPs showed higher n values.

**Figure 7:**
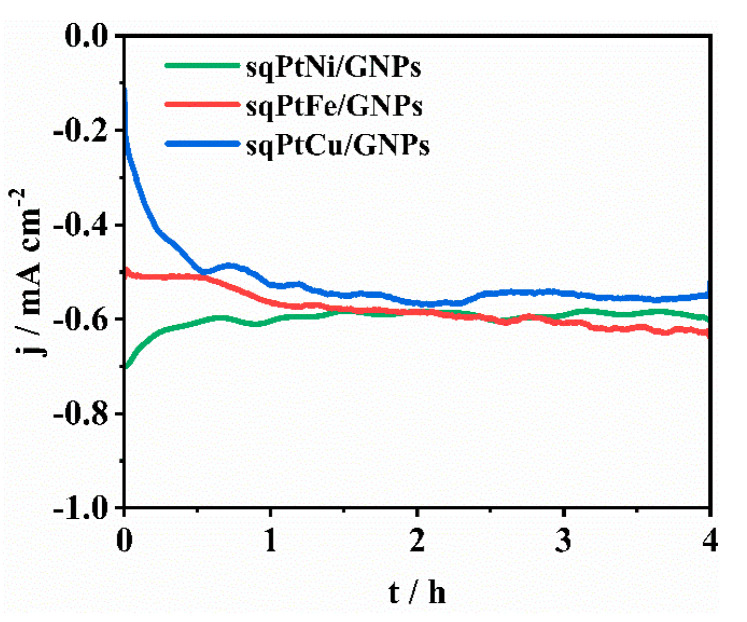
ORR stability test of the catalysts in O_2_-saturated 0.1 M KOH at 0.6 V vs. RHE.

Figure 7 presents the stability test results for the three prepared catalysts performed in chronoamperometric mode at 0.6 V for 4 h with constant O_2_ bubbling. Catalysts showed relatively stable currents during experiments. Small fluctuations originated from O_2_ bubbling, which mechanically disturbs the electrode/electrolyte solution interface, causing current oscillations. In the case of sqPtCu/GNPs and sqPtFe/GNPs, the current density slightly increased with time, suggesting activation of the catalyst material.

### 3.4. OER Study

The bifunctional performance of the synthesised electrocatalysts as URFCs or metal-air batteries’ positive electrodes was assessed by investigating their catalytic activity towards OER recording LSVs at 1200 rpm. These experiments are performed after ORR measurements with the same physical electrode in the same cell.

**Figure 8:**
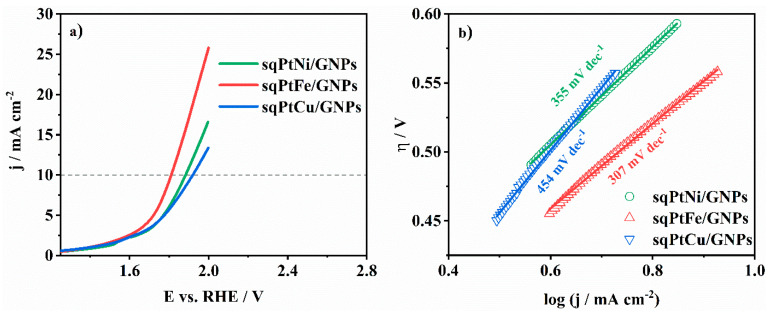
(**a**) LSVs (IR-corrected) of the three sqPtM/GNPs electrocatalysts in 0.1 M KOH with (**b**) the corresponding Tafel plots.

Once again, sqPtFe/GNPs showed the highest current density and the lowest overpotential to achieve a current density of 10 mA cm^−2^, η_10_, (0.582 V), as shown in Figure 8a. η_10_ values were somewhat higher for sqPtNi/GNPs and sqPtCu/GNPs (0.654 V and 0.690 V, respectively). This trend is also observed when assessing current density at an overpotential of 400 mV. Namely, sqPtFe/GNPs gave the highest value of j_400_ (2.98 mA cm^−2^) followed by the sqPtCo/GNPs and sqPtNi/GNPs samples.

A key parameter to assess the bifunctional capacity of an electrocatalyst is the potential difference, ΔE, between the ORR half-wave potential and the overpotential to achieve a current density of 10 mA cm^2^ in OER mode. For sqPtFe/GNPs, ΔE was calculated to be 0.912 V, comparable to or even lower than some literature data involving Fe-based catalysts [19,21]. Table 2 presents the calculated parameters for studied electrocatalysts and compares them with the latest relevant literature data.

The promising catalytic performance of the samples towards oxygen electrode reactions in URFCs or metal-air batteries was further confirmed by performing Tafel analyses (Figure 8b). Once more, sqPtFe/GNPs showed the lowest Tafel slope (307 mV dec^−1^), followed by the sqPtNi/GNPs and sqPtCu/GNPs samples, with Tafel slopes of 355 and 454 mV dec^−1^, respectively.

**Figure 9:**
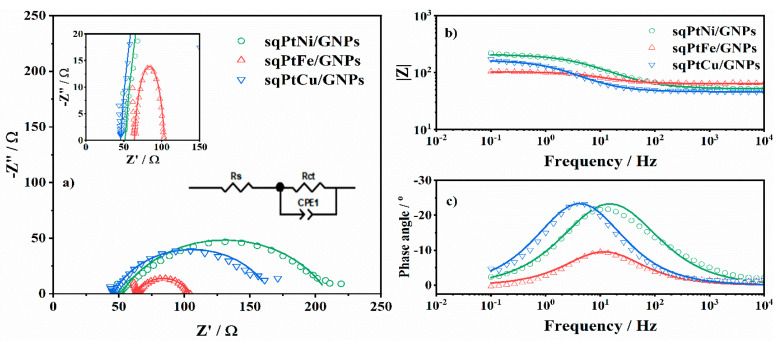
(**a**) Nyquist plots with the zoomed-in plot of sqPtFe/GNPs and equivalent circuit used to fit the EIS data in the inset, and (**b**,**c**) Bode plots of the three electrocatalysts.

Electrochemical impedance spectroscopy (EIS) data, shown in Figure 9, were fitted using the equivalent circuit presented as an inset of Figure 9a. R_s_, the high-frequency intersection of the spectra with the Z’-axis of the Nyquist plot, represents the sum of the resistances of the electrode material, wiring, and resistance of the electrolyte. Furthermore, the flattening of the semicircle in the Nyquist plots indicates dispersive capacitance [29]. The constant phase element (CPE) is used as an alternative to pure capacitance, accounting for surface heterogeneity or distribution of time constants on the electrode surface [30]. One parallel R_ct_-CPE element is used in series with R_s_ to describe the charge transfer process. Charge transfer resistance, R_ct_, related with the overall reaction rate, accounts for the charge transfer resistances of the various steps [31].

EIS studies revealed that sqPtFe/GNPs sample has the lowest R_ct_ value of ≈40 Ω. Namely, significantly higher R_ct_ values of ≈158 Ω and 120 Ω were observed for sqPtNi/GNPs and sqPtCu/GNPs, respectively. All EIS fit parameters are presented in Table 3.

**Figure 10:**
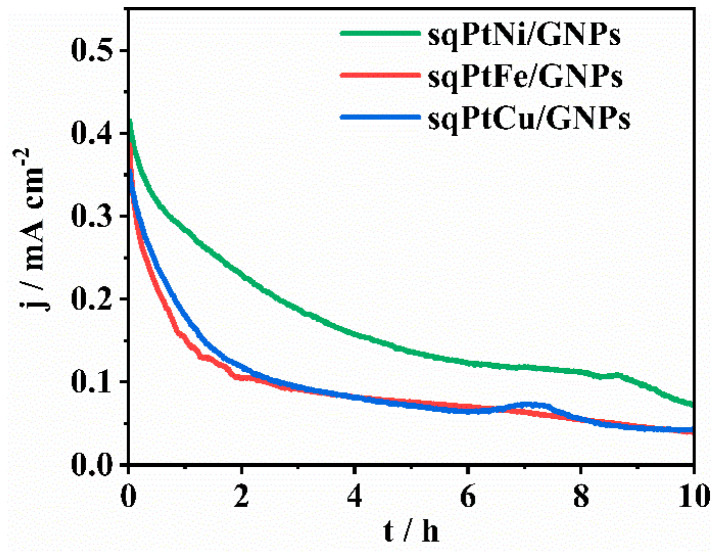
OER stability tests in “switching mode” at 1.8 V vs. RHE (only OER current densities are shown).

Figure 10 presents the stability test results for the three studied electrocatalysts in “switching mode”. For simplicity, only the OER part of the curves is shown. A significant drop in current densities is observed in the first 2 h of testing, after which currents are relatively stable but still show a decreasing trend. It should be noted that intense bubbling was observed when the catalyst operated in OER mode. This may lead to partial catalysts’ detachment from the glass carbon disc electrode, resulting in a current drop. Stability may be increased by optimising the catalyst ink preparation and changing the Nafion binder.

The Fe-based catalyst showed the most promising bifunctional activity among the studied catalysts. Fe-based electrocatalysts have been suggested as appropriate alternatives among non-PGM (platinum group metal), transition metal-based electrocatalysts for ORR in alkaline media, being abundant and highly efficient [32,33,34,35]. However, reports on the activity of Fe-based electrocatalysts for the OER and thus for bifunctional ORR/OER performance, are rather scarce [36,37]. One study reports the bifunctional behaviour of Fe (and other transition metals) supported on N-doped porous carbon [38]. Still, these materials are typically produced by high-temperature methods, for instance, pyrolysis of metal-organic frameworks [34,35], during which metal species’ sintering and/or aggregation occurs. This poses an obstacle in obtaining a material with a homogeneous distribution of active sites [39]. However, the sequential method used herein resulted in materials with a homogeneous distribution of metal NPs over the carbon support. Furthermore, electrocatalysts obtained via the sequential deposition method showed better electrocatalytic behaviour for ORR and OER than the corresponding electrocatalysts obtained by simultaneous deposition, as shown in Table 1 and Table 2.

The beneficial effect of alloying Pt with Fe [40] results from changes in Pt geometric and electronic structures, including alterations of the Pt-Pt bond distance, Pt 5d vacancy, and d-band centre. These changes further lead to changes in the adsorption of intermediate oxygen species and thus influence the ORR activity to a great extent [5]. First, Fe acts as an adsorption site for “capturing” oxygen and increases adsorption capacity towards oxygen, leading to a decrease of the adsorption Gibbs energy. The interaction between Fe and Pt alters the Pt-Pt bond distance. Fe causes the lattice contraction reducing the interatomic distance that governs the adsorption and transfer of oxygen-containing species. Finally, the alterations of overlap of orbital and the electronic properties on the active site by lattice contraction alter the surface reactivity.

The presence of defects (e.g., point, line, plane, or volume defects) in the crystal structure results in the variation of material properties, including electrical and chemical activity [41,42]. The local electronic state of the catalyst can be redistributed, affecting the adsorption energy of the intermediate species. In the case of Pt alloys, step edge has been reported to reduce the d-band centre, resulting in facilitated desorption of the intermediate species and thus in enhanced ORR activity. Furthermore, the presence of a large quantity of disordered/defective carbon was also revealed by Raman analysis, with the presence of such carbon boosting the materials’ catalytic activity towards the ORR due to the higher number of active sites [4,43].

The materials’ structure is essential for their electrochemical activity as well as for their stability under polarisation conditions, as demonstrated for unsupported Pt-Fe NPs [40]. High stability has been observed under the ORR conditions and lower stability under the OER conditions. However, the authors believe that this is a consequence of film falling off the conductive tip rather than metal alloy NPs being detached from the carbon support.

Thus, a rather simple but efficient approach to loading metal alloy NPs onto carbon substrate that provides exposure of reaction sites and promotes charge transport by decreasing the diffusion length of electrolytes and gases was presented herein [44]. Furthermore, the observed high stability under ORR polarisation conditions provides evidence that such a structure prevents metal NPs from agglomerating or leaching. Therefore, studies using PtFe/GNPs in a zinc-air battery are underway.

## 4. Conclusions

Three alloys (PtNi, PtFe, and PtCu) on GNPs were synthesised by sequential deposition via supercritical carbon dioxide. Metal NPs of 3–5 nm diameter were evenly distributed over the GNPs. Out of the three materials tested, sqPtFe/GNPs exhibited the highest activity for both the ORR and OER, with a potential difference of 0.912 V between the half-wave potential of ORR and the OER potential at 10 mA cm^−2^. sqPtFe/GNPs exhibited better activity compared to the commercially available Pt/C, which contains twice the amount of Pt. Moreover, negligible decay of ORR current density with time was observed. Thus, the sequential deposition method was demonstrated to be a rather simple but efficient approach to loading metal alloy NPs onto a carbon substrate that provides exposure of reaction sites and promotes charge transport. Excellent ORR/OER performance of sqPtFe/GNPs with the employment of a reduced amount of noble metal suggests that this material has many electrochemical energy conversion applications.

## Figures and Tables

**Table 1:** Key ORR parameters for catalysts studied herein, and catalysts reported in the literature.

Material	b_1_/ mV dec^−1^	b_2_/ mV dec^−1^	j_d_/mA cm^−2^	n	j_k_/mA cm^−2^	C_dl_/mF cm^−2^	ECSA/cm^2^	E_1/2_ vs. RHE/V	Source
sqPtNi/GNPs (sequentially)	106	-	−4.14	4.00	−2.41	7.9	197.5	0.98	This work
sqPtFe/GNPs (sequentially)	100	-	−4.48	3.85	−3.65	5.6	140	0.90	This work
sqPtCo/GNPs (sequentially)	105	-	−3.99	3.83	−2.51	7.5	187.5	0.92	This work
Pt/GNPs	65	102	−3.71	3.77	−2.05	8.2	205	0.98	[5]
PtNi/GNPs (simultaneously)	102	-	−3.65	3.93	−1.73	4.5	112.5	0.94	[5]
PtFe/GNPs (simultaneously)	81	66	−4.65	3.66	−5.94	7.2	180	0.91	[5]
PtCu/GNPs (simultaneously)	67	-	−4.37	3.61	−3.69	5.1	127.5	0.90	[5]
Pt/C (40 wt.% Pt)	79	60	−6.43	3.97	14.9	3.10	77.5	-	[8]
Fe-N_x_-C	69	-	−5.44	3.9	14.7	-	-	0.91	[19]
Fe-N/C-700	-	-	≈−5.9	4.02	4.1	-	-	0.84	[20]
Fe-N/C	92.5	-	≈−5.9	-	-	20.06	-	0.818	[21]
FeCu_0.3_-N/C	92.1	-	≈−6.1	-	-	27.74	-	0.865	[21]
7.5Pd-2.5Cu	-	-	≈−8	4.1	-	-	-	≈0.90	[22]
7.5Pd-2.5Fe	-	-	≈−8.4	3.9	-	-	-	≈0.83	[22]
7.5Pd-2.5Ni	-	-	≈−9	4.1	-	-	-	≈0.86	[22]
IrP_2_@PC	-	-	≈−6	3.98	-	-	-	0.81	[23]
Ru_1.0_/HNCS	107	-	≈−4.2	3.97	-	-	-	-	[24]

b—Tafel slope; j_d_—diffusion-limited current density; n—number of exchanged electrons in the elementary step; j_k_—kinetic current density; C_dl_—double-layer capacitance; ECSA—electrochemically active surface area; E_1/2_—half-wave potential. HNCS—hollow nitrogen-doped carbon spheres.

**Table 2:** Key OER kinetic parameters of the catalysts tested herein, and catalysts reported in the literature.

Material	b/mV dec^−1^	j_0_/mA cm^−2^	η_10_/V	j_400_/mA cm^−2^	ΔE/V	Source
sqPtNi/GNPs (sequentially)	355	0.186	0.654	2.47	0.904	This work
sqPtFe/GNPs (sequentially)	307	0.143	0.582	2.98	0.912	This work
sqPtCo/GNPs (sequentially)	454	0.268	0.690	2.59	1.00	This work
Pt/GNPs	478	0.286	0.765	1.95	1.02	[5]
PtNi/GNPs (simultaneously)	356	0.187	0.652	2.64	0.94	[5]
PtFe/GNPs (simultaneously)	280	0.118	0.572	2.89	0.89	[5]
PtCu/GNPs (simultaneously)	490	0.295	0.660	3.27	0.99	[5]
Pt/C (40 wt.% Pt)	198	0.030	0.576	1.59	-	[8]
Fe-N_x_-C	243	-	0.600	-	0.92	[19]
NiFe_2_O_4_	35.9	-	0.326	-	-	[25]
Fe-N/C	334.4	-	0.545	-	0.957	[21]
FeCu_0.3_-N/C	263.4	-	0.412	-	0.777	[21]
IrO_2_@CNT	44	-	0.217	-	-	[26]
CoCrRu LDHs	56	-	0.290	-	-	[27]
RuCo@NC-750	140	-	0.308	-	-	[28]

CNT—carbon nanotubes; LDHs—layered double hydroxides.

**Table 3:** EIS fit parameters for three tested electrocatalysts.

Parameter	Material		
	sqPtNi/GNPs	sqPtFe/GNPs	sqPtCu/GNPs
R_s_/Ω	52 ± 1%	64 ± 1%	46 ± 1%
R_ct_/Ω	158 ± 2%	40 ± 5%	120 ± 4%
Q_1_/s^N^ Ω^−1^	0.00053 ± 7%	0.00115 ± 22%	0.00137 ± 8%
N_1_	0.70 ± 2%	0.78 ± 7%	0.75 ± 3%

## Data Availability

The data presented in this study are available on request from the corresponding author.
